# Effect of Copper Surface Roughness on the High-Temperature Structural Stability of Single-Layer-Graphene

**DOI:** 10.3390/ma17071648

**Published:** 2024-04-03

**Authors:** Songsong Yao, Boan Zhong, Chongxiao Guo, Jiamiao Ni, Kunming Yang, Siqi Hu, Zheng Gong, Yue Liu, Jian Song, Tongxiang Fan

**Affiliations:** 1 State Key Lab of Metal Matrix Composites, School of Materials Science and Engineering, Shanghai Jiao Tong University, Shanghai 200240, China; 2 Institute of Materials, China Academy of Engineering Physics, Mianyang 621900, China

**Keywords:** copper, surface roughness, graphene, high-temperature structural stability

## Abstract

Graphene (Gr) has shown great potential in the field of oxidation protection for metals. However, numerous studies have shown that Gr will suffer structural degradation on metal surface during high-temperature oxidation, which significantly limited the effectiveness of their oxidation protection. Therefore, understanding the degradation mechanism of Gr is of great interest to enhance their structural stability. Here, the effect of copper (Cu) surface roughness on the high-temperature structural stability of single-layer graphene (SLG) was examined using Cu covered with SLG as a model material. SLG/Cu with different roughness values was obtained via high-temperature annealing of the model material. After high-temperature oxidation at 500 °C, Raman spectra analysis showed that the defect density of the oxidized SLG increased from 41% to 81% when the surface roughness varied from 37 nm to 81 nm. Combined with density functional theory calculations, it was found that the lower formation energy of the C-O bond on rough Cu surfaces (0.19 eV) promoted the formation of defects in SLG. This study may provide guidance for improving the effectiveness of SLG for the oxidation protection of metallic materials.

## 1. Introduction

Metallic materials such as copper (Cu) and silver have been widely used in electric industries because of their excellent electrical conductivity [1,2,3]. However, these materials always exhibit high chemical reactivity at elevated temperatures, which can lead to severe surface oxidation and consequently degrade the electrical conductivity and lifetime of electronic devices [4,5,6]. Therefore, improving the surface oxidation resistance has been one of the core topics in the study of metallic materials for decades [7,8]. Generally, enhancing the surface oxidation resistance of metals can be achieved by tailoring the surface crystallography [9,10], alloying the surface [11,12] or introducing surface coatings [13,14]. Compared with other strategies, surface coatings can physically isolate the metal surface from the oxygen environment, thereby significantly enhancing surface oxidation resistance. Correspondingly, various coating technologies, such as vapor deposition [15], spray coating [16] and plasma electrolytic oxidation [17,18], have been developed to synthesize various coatings (including metallic and ceramic) on metal substrates. Moreover, extensive studies also suggest that the oxidation resistance of coatings can be tailored by controlling their chemical composition [19], microstructure [20] and adhesion strength on a metal matrix (interface) [21,22]. Despite these efforts, the harsh working conditions (high frequency and high-power density) of modern electric industries require a synergetic enhancement of both surface oxidation resistance and electrical conductivity. In order to meet the requirement of high-performance components in harsh working conditions, superior coating with high conductivity and excellent oxidation resistance, etc., need to be accurately developed.

Graphene (Gr), with a thickness on the order of nanometers, may serve as a very promising coating material owing to its excellent structural stability, high oxygen barrier and good electrical conductivity [23,24]. Extensive research has demonstrated the enhanced electrical conductivity of Gr coated on Cu surface [25,26,27], in comparison to the pure Cu counterpart. For example, Cao deposited Gr on single-crystal (111) Cu foils and followed with hot pressing to obtain a Gr/Cu composite with a Gr volume fraction of 0.008%. The four-probe test results showed that the conductivity of the Gr/Cu conductor was up to nearly 117% International Annealed Copper Standard (IACS) [25]. Kashani deposited Gr on Cu wires with nominal diameters of 10 μm, 25 μm and 80 μm, respectively. It was found that the resistivities of 80 μm, 25 μm and 10 μm wires were 4%, 22% and 41% lower than their annealed counterparts [26]. Mehta prepared Cu nanowires (NMs) by employing e-beam lithographical patterning and argon sputter etching on physically vapor-deposited Cu films with the thickness of 60 nm. Gr was subsequently deposited on the surface of Cu NMs using plasma-enhanced chemical vapor deposition (CVD) with methane (CH_4_) as the carbon source. Electrical test results showed that the resistance of Cu NMs with a Gr coating was nearly 15% lower than that of the counterparts without Gr [27]. All of these findings demonstrate the great potential of Gr in enhancing the electrical conductivity of metallic materials.

However, the effectiveness of Gr for enhancing the surface oxidation resistance of metals is significantly different from expectations. For instance, Chen used CH_4_ as a carbon source to prepare Gr on the surface of a Cu foil via CVD at 1040 °C. The samples were then annealed in air at 200 °C for 4 h. Compared to the pure Cu counterparts, the deposited Gr manifested excellent protection against oxidation at high temperatures [28]. Song deposited Gr with mixed number of layers on the Cu surface at 1045 °C and found that Gr can provide oxidation protection to Cu at 550 °C for 20 min. However, when increasing oxidation time, it was found that oxygen can diffuse through the defects and grain boundaries (GBs) of Gr, thereby leading to the severe oxidation of the Cu substrate [29]. Meanwhile, Schriver reported that Gr is an effective antioxidation barrier on Cu during short-term (on the time scale of minutes) thermal oxidation testing (185 °C). After prolonged thermal exposure, the defects and grain boundaries (GBs)of Gr can act as diffusion channels for oxygen, initiating inhomogeneous Cu oxidation and promoting the structural failure of the Cu matrix [30]. In addition, the number of Gr layers also affects the oxidation behavior of the Cu surface. Luo studied the oxidation rate of the Gr-coated Cu {111} surface and found that the oxidation rate in the bilayer Gr (BLG) covered region was considerably slower than that covered with single-layer Gr (SLG). Combined with density functional theory (DFT) simulations, Luo attributed the phenomenon to the lower energy barrier required for oxygen to diffuse through SLG [31]. Apart from oxygen diffusion associated with defects/GBs and layer numbers of Gr, it was found that the structural stability of Gr can also determine the anti-oxidation properties of Gr/Cu. Song studied the oxidation resistance of SLG/Cu. It was found that the Raman G peak of SLG nearly vanished after oxidation at 550 °C for 30 min, suggesting that severe structural damage occurred in Gr. The formation of structural damage in Gr can lead to the degradation of surface oxidation resistance of Gr/Cu. These studies suggest that the profuse diffusion channels of oxygen (due to defects, GBs) and low stability of Gr on Cu surfaces are the reasons for the severe oxidation of Gr-coated Cu. Considering that the defects/GBs, density and number of layers can be tailored by controlling the synthesis parameters of Gr, improving the stability of Gr may further enhance the oxidation resistance of Gr-coated metals. Correspondingly, a prerequisite is to understand the degradation mechanism of Gr on metal surface.

For Gr-coated metals, the stability of Gr lies in the substrate material properties, such as grain size, surface orientation and surface roughness. Especially surface roughness plays an important role in the nucleation and growth stages of Gr, it might have the most profound effect on the structural integrity and stability of Gr. Extensive research has been conducted to investigate the effect of substrate on Gr structure [32,33,34,35]. For example, Fan deposited Gr on Cu surfaces with roughness of 0.074–0.339 μm, and the results showed that substrate roughness seriously affected the grain size and quality of Gr [32]. Wang studied the effect of Cu substrate roughness on Gr domains and pointed out that Cu with a flat surface is beneficial for the growth of large size single-crystal Gr [33]. Wofford prepared ultra-smooth nickel (Ni) films on the surface of MgO substrates using molecular beam epitaxy. Gr was then deposited on the Ni surface, and structural characterization revealed that the Gr had few crystalline defects [34]. Procházka prepared Gr via CVD on the surface of sputter-deposited Cu films and found that Gr synthesized on Cu surfaces with roughness of 0.6 nm was of high quality and demonstrated ultra-high room-temperature carrier mobility [35]. All these studies demonstrated that substrate roughness can severely affect the structural integrity of Gr. 

Although these advances may greatly enhance the application of Gr, it remains to be elucidated that the influence of surface roughness on the surface oxidation process. Generally, rough metal surfaces consist of large facets/steps, which always serve as active sites to promote surface oxidation [36,37]. Gr coatings can be commensurate with surface morphology and isolate these facets/steps from the surrounding environment, but their electronic structures are inevitably changed owing to the local bending strain of Gr at these facets/steps [38,39]. Given that the local bending strain can alter the chemical activities of most materials, it is believed that surface roughness can also change the antioxidation properties of Gr. However, the effect and mechanism of surface roughness on the antioxidation of Gr coatings remain ambiguous: while some studies suggest that metal surface roughness can promote the oxidation of Gr/Cu, others show that this has virtually no influence on their antioxidation behavior [40,41]. In order to expand the full potential of Gr for surface protection and develop corresponding strategies to fabricate Gr coatings on metal, a comprehensive study is required to elucidate the effect and mechanism of surface roughness on the antioxidation properties of Gr coatings.

To this end, this paper proposes to carry out a study on the effect of substrate roughness on the high-temperature structural stability of Gr. It is important to prepare Gr-coated metal samples with similar Gr structures and different metal surface roughness. Recent studies [42,43,44] have demonstrated that high-temperature annealing of the Gr/Cu bilayer can tailor the height and density of surface steps and is ultimately reflected in the average surface roughness (Ra) of Cu. In addition, in order to exclude the effect of Gr layer number, single-layer graphene (SLG) was chosen as the model Gr material. In addition, the non-mixing nature of SLG and Cu also makes SLG/Cu an ideal model to further reveal the effect of surface roughness on the structural degradation of SLG. In this paper, SLG/Cu foils were annealed at 600 °C for 30, 60 and 90 min to obtain SLG/Cu bilayers with substrate roughness values of 81, 45 and 37 nm, respectively. After accelerating oxidation via high-temperature treatment (500 °C under an O_2_ atmosphere), Raman spectroscopy and X-ray photoelectron spectroscopy (XPS) results showed that the density of C-O defects doubled as the annealing duration increased from 30 min to 90 min, indicating that significant surface roughness can degrade the thermal stability of SLG. Combined with DFT calculations, this provides evidence that the formation energy of C-O is significantly reduced at large surface steps, which further confirms that the surface roughness of metals can determine the antioxidation behavior of SLG. This study not only reveals the degradation mechanism of SLG on a metal surface, but also suggests that the oxidation resistance of coatings (SLG) can be improved by tailoring the surface roughness of the metal substrate.

## 2. Materials and Methods

### 2.1. Materials and Characterizations

Pristine Cu foils with a thickness of ~25 μm and a width of ~60 mm (KJMTI Corp., Hefei, China) were introduced to a roll-to-roll low-pressure chemical vapor deposition (CVD) system (homemade equipment) with a quartz furnace tube 1.5 m in length and 80 mm in diameter. Then the pressure was reduced to below 10^−2^ mTorr, hydrogen (H_2_) gas (Airliquide Co., Ltd., Shanghai, China) was introduced into the furnace at a flow rate of 200 standard cubic centimeters per minute (sccm) and the quartz furnace tube was heated up to 1050 °C at a rate of 20 °C/min. Subsequently, the Cu foils were held at 1050 °C for 30 min to completely remove the oxides and impurities from the surface of the Cu. After that, methane (CH_4_, Airliquide Co., Ltd., Shanghai, China) was introduced into the furnace tube at a flow rate of 6 sccm to grow the SLG, and the duration was kept to 15 min. After SLG deposition, the samples were pulled into the water-cooling system by the roll-to-roll mechanism and cooled down at a cooling rate of up to 20 °C/s. When the temperature of the Cu foils dropped below 50 °C, the injection of H_2_ and CH_4_ stopped. Next, the as-deposited SLG/Cu was cut into three pieces for tailoring roughness. The SLG/Cu foil was placed in the quartz furnace tube, and the pressure was reduced to below 10^−2^ mTorr. Argon (Ar, Airliquide Co., Ltd., Shanghai, China) was then introduced into the furnace tube and the pressure was maintained at 800 Torr. The CVD system was then heated to 600 °C at a rate of 20 °C/min, and the pressure was held at ~800 Torr during the heating process. In order to obtain SLG/Cu foils with various surface roughness values, the three pieces of SLG/Cu foil were annealed at 600 °C (A_d_) for 30, 60 and 90 min, respectively. Correspondingly, these samples were labeled A-30, A-60 and A-90, respectively. After that, high-temperature oxidation was performed in a muffle furnace under an O_2_ environment at 500 °C for 60 s. The oxidized samples were labeled O-A-30, O-A-60 and O-A-90, respectively. The details of the designed samples are shown in Table 1.

The average Ra of SLG/Cu samples was analyzed using confocal optical microscopy (COM, RX-100, Hirox Co., Ltd., Tokyo, Japan). The laser wavelength in the test was 485 nm and the magnification was 500×. Minimum of 3 tests were performed on 3 specimens for each parameter to obtain an average surface roughness. Further surface step features were characterized via scanning electron microscopy (SEM, RISE-Magna, TESCAN, Brno, Czech). Before and after the oxidation treatment, the Raman spectrum and mapping results were obtained using Raman spectroscopy (InVia Reflex, Renishaw, London, UK) with a 532 nm Ar+ laser at ambient temperature. In the Raman spectrum test, the parameters were set to a laser power of 60 mW, a scan time of 10 s for 5 repetitions with an acquisition wavenumber range of 1100–3100 cm^−1^. In the Raman mapping test, the parameters were set to a laser power of 100 mW, a step size of 1 μm and a scanning area of 10 × 10 μm^2^. The defect types of graphene were tested via X-ray photoelectron spectroscopy (XPS, Nexsa, Worcester, MA, USA), using monochromaticized Al Kα as the X-ray source at a test power of 150 W, scanning step size of 1000.0 meV and residence time of 100 ms. The full spectrum scanning range was 0–1200 eV, while the C 1 s spectrum scanning ranges were 281–291 eV. 

### 2.2. Density Functional Theory (DFT) Calculations

The DFT calculations were conducted using the Vienna Ab initio Simulation Package (VASP 5.4.4) [45]. The generalized gradient approximation (GGA) with Perdew–Burke–Ernzerhof (PBE) [46] parametrization and empirical vdW correction (DFT-D3) [47] was used for the exchange and correlation functions. The interaction between valence electron and ion cores was described via projector augmented wave (PAW) [48], following the convergence of the cut-off energy, k-mesh, and the number of atomic layers. A 450 eV cut-off for kinetic energy was used for the plane-wave basis set. For all DFT calculations, the self-consistent iteration was stopped when the change in total energy was smaller than 10^−5^ eV. The convergence criterion of geometry optimizations was that the force acting on each atom was smaller than 0.02 eV/Å. Γ-centered grids of 15 × 15 × 1, 15 × 15 × 15 and 2 × 4 × 1 were used for Gr, Cu and Gr/Cu models, respectively. After optimization, the calculated lattice constants of Cu and Gr were 3.56 Å and 2.47 Å, which agrees well with experiments [49]. A 4√3× 12 × 1 supercell model consisting of four Cu (111) layers and a single layer of Gr (comprising 128 Cu atoms and 56 C atoms) was established with the bottom layer of Cu atoms fixed. A small ~2% strain was applied to the Cu substrate to maintain coherency at the interface. To ensure the artificial interaction between the interface in adjacent cells, a vacuum spacing of at least 15 Å was set in the supercell along the c-axis; the dipole correction [50] was not considered due to its negligible impact in this study. Additionally, when considering the influence of Cu edges, half of the atoms of the top Cu layer was removed. The edges of the Gr were hydrogenated to prevent interaction between carbon (C) atoms along the edges of the Gr and Cu steps. VASPKIT [51] was used for pre-/post-processing, and VESTA [52] was used for visualization.

The adsorption energy $E_{ads}$ at the interface was calculated as follows:

(1) $$E_{ads}=E_{O-SLG/Cu}-E_{SLG/Cu}-E_{O}$$

where $E_{O-SLG/Cu}$, $E_{SLG/Cu}$ and $E_{O}$ were the energy for SLG/Cu with oxygen, SLG/Cu and an oxygen (O) atom, respectively.

## 3. Results

**Figure 1:**
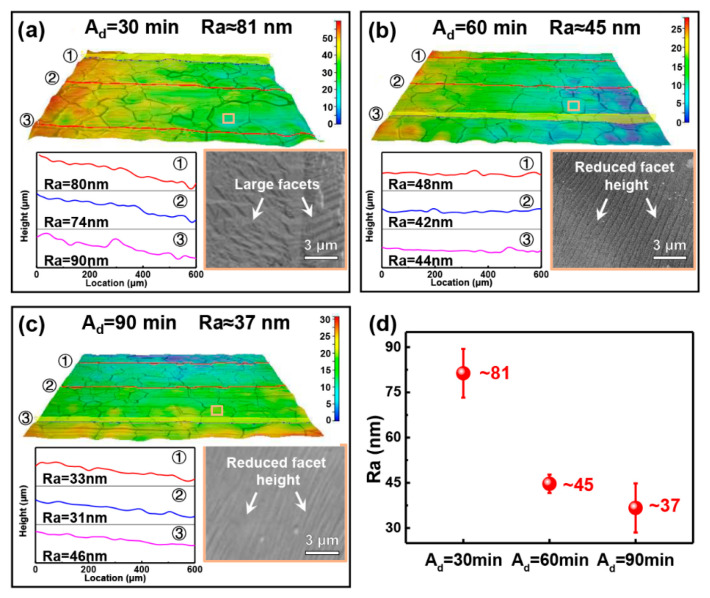
Surface roughness characterization results of Cu samples covered with SLG treated for different annealing durations (A_d_). (**a**) A_d_ = 30 min; (**b**) A_d_ = 60 min; (**c**) A_d_ = 90 min; (**d**) variation in roughness (Ra) as a function of annealing duration.

Figure 1 compares the surface roughness of Cu surfaces following different annealing durations. The COM images in Figure 1a–c show that both samples exhibit a polycrystal structure, and the grain sizes are comparable. To ensure the obtained Ra is representative, a minimum of three roughness curves were measured on each sample, and the average value was calculated as Ra of the SLG/Cu foils. It was found that the surface Ra decreased with increasing annealing duration (A_d_). Specifically, the Ra decreased from 81 nm to 37 nm as the A_d_ ranged from 30 min to 90 min (Figure 1a–c). The appearance of surface variation, i.e., changes in the density and height of the surface steps, may occur during the annealing process. Further SEM analysis (the insets on the lower right of Figure 1a–c) confirmed the variation in the surface steps and showed that the densities and heights of the surface steps decreased with increasing A_d_. The mechanism underlying the step evolution can be attributed to the minimization of SLG strain energy, which has been discussed in the authors’ previous work [43]. Therefore, it is possible to tailor the surface roughness of SLG-coated Cu foils using the SLG-assisted surface reconstruction.

**Figure 2:**
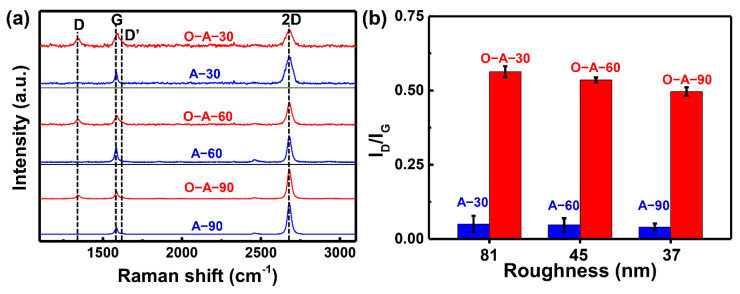
Comparison of (**a**) Raman spectroscopy and (**b**) I_D_/I_G_ results before (blue) and after (red) high-temperature oxidation treatment of SLG on Cu with different surface roughness values.

Subsequently, Raman spectra were utilized to characterize the microstructures of SLG in these annealed samples. Figure 2a shows the comparison of Raman spectroscopy results of annealed SLG/Cu (blue). It was found that there are only G peaks located near 1580 cm^−1^ and 2D peaks near 2680 cm^−1^ [53]. In addition, I_2D_/I_G_ was larger than 2, indicating that the deposited Gr was still SLG [53]. Also, the Raman spectra showed no difference in peak shapes, intensities and positions, suggesting that the annealing treatment under the protective atmosphere has no obvious effect on the SLG structure. Moreover, these results also demonstrate that the SLG on these samples is comparable, which largely ruled out the influence of surface roughness on the SLG structure. Therefore, the evolution of Raman peaks can be used to characterize the SLG structure after oxidation testing. After the high-temperature oxidation treatment, the samples were characterized via Raman spectroscopy and the results are shown in Figure 2a. Compared to the annealed SLG/Cu, D peaks appeared at ~1350 cm^−1^, and D’ peaks presented at ~1620 cm^−1^ [54], indicating that the structure of SLG had severely deteriorated after high-temperature oxidation. To quantitatively analyze the characteristic peaks in the Raman spectra, the I_D_/I_G_ ratios in Figure 2a were calculated and plotted in Figure 2b. Before the oxidation treatment, the I_D_/I_G_ ratios are both lower than 0.1 (blue), suggesting that the defects are the intrinsic defects of SLG [55]. Additionally, this result also shows that the number of defects before the oxidation treatment is on the same order of magnitude, thus further confirming that the quality of SLG is comparable among the annealed samples. After oxidation treatment, the I_D_/I_G_ ratios of the O-A-30, O-A-60 and O-A-90 samples were 0.56, 0.54 and 0.50, respectively. Since the I_D_/I_G_ value can reflect the density of the SLG defect, these results suggested that (1) a large number of defects appeared in the SLG after oxidation treatment and that (2) the density of SLG defects decreases with decreasing of surface roughness.

**Figure 3:**
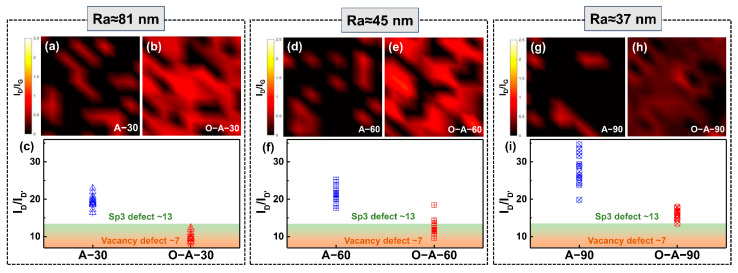
Raman mapping results of SLG. I_D_/I_G_ mapping results of SLG/Cu before high-temperature treatment for samples Ra values of (**a**) Ra ≈ 81 nm, (**d**) Ra ≈ 45 nm and (**g**) Ra ≈ 37 nm, respectively; I_D_/I_G_ mapping results of SLG/Cu after high-temperature treatment for samples with Ra values of (**b**) Ra ≈ 81 nm, (**e**) Ra ≈ 45 nm and (**h**) Ra ≈ 37 nm, respectively. Comparison of I_D_/I_D’_ values for 15 points randomly selected from (**c**) a and b, (**f**) d and e and (**i**) g and h, respectively.

**Figure 4:**
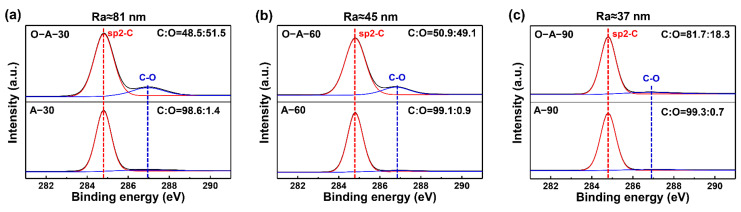
XPS characterization results of SLG. (**a**) XPS results of a sample for which Ra ≈ 81 nm before (A-30) and after (O-A-30) high-temperature treatment; (**b**) XPS results of a sample for which Ra ≈ 45 nm before (A-60) and after (O-A-60) high-temperature treatment; (**c**) XPS results of a sample for which Ra ≈ 37 nm before (A-90) and after (O-A-90) high-temperature treatment.

In order to ensure the Raman results are representative, Raman mapping was used to characterize the SLG structure before and after oxidation treatment. The results are shown in Figure 3. The color represents the I_D_/I_G_ ratio and the values can reflect the density of the SLG defect. Therefore, the distribution and values of the colored region can be used to visualize the distribution and density of the defects in SLG. By comparing the Raman mapping results before and after oxidation treatment, it was found that the I_D_/I_G_ ratios had increased significantly after oxidation treatment (Figure 3a,b,d,e,g,h). Besides, the I_D_/I_G_ ratio in the oxidized SLG decreased with increasing Ra, which is consistent with the results in Figure 2b and previous literature [54]. In addition to I_D_/I_G_ ratios, I_D_/I_D’_ can be used to determine the type of defect in SLG [56]. It has been reported that the SLG defect is of the sp3 type when I_D_/I_D’_ ≈ 13, as compared to the vacancy type when I_D_/I_D’_ ≈ 7. Based on this, 15 points were randomly selected from the Raman mapping results, and the I_D_/I_D’_ peak ratios were calculated and plotted as scatter plots in Figure 3c,f,i. It was found that the average I_D_/I_D’_ in Figure 3a,d,g exceeded 20, indicating the near absence of defects in the annealed samples. After oxidation testing, the average values of I_D_/I_D’_ in O-A-30/O-A-60/O-A-90 samples were ~10, ~12 and ~16, respectively, suggesting the presence of defects and the main defects are of the sp3 type [56]. To further confirm the density of sp3 defects in SLG, XPS characterizations were performed and the results are shown in Figure 4. It was shown that there are two characteristic peaks located at 284.8 eV (sp2C, red curve) and 286.8 ± 0.2 eV (C-O, blue curve) [57] in the oxidized samples. By comparison, the C-O peaks of the untreated samples were not observed, and their O-atom ratios were found to be below 1.5. It is worth noting that the appearance of O in A-30, A-60 and A-90 samples may be attributed to the inevitable exposure of these samples to the atmospheric environment during testing. After oxidation treatment, the intensity of the C-O peaks increased significantly, corresponding to C:O ratios of 48.5:51.5, 50.9:49.1 and 81.7:18.3, respectively. Specifically, a lower Ra value can lead to lower O content and fewer C-O bonds in the oxidized SLG. Therefore, these results not only verified that the defects are primarily of the sp3 type, but also suggest that the structural stability of SLG may degrade with increasing Ra.

## 4. Discussion and Conclusions

**Figure 5:**
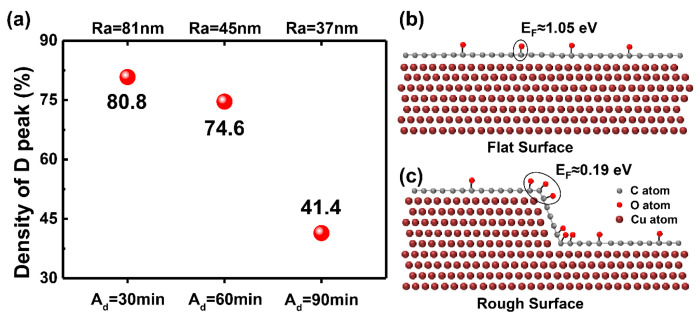
D-peak density and DFT results for SLG/Cu. (**a**) D-peak density in Raman mapping and formation energy of the C-O bond on (**b**) flat and (**c**) rough Cu surfaces covered with SLG.

In order to elucidate the variation of SLG defects with Ra, the density of the D peak was used to qualitatively describe the defect density in SLG and the results were plotted as a function of Ra. As shown in Figure 5a, the densities of the D peaks in O-A-30, O-A-60 and O-A-90 samples were 80.8%, 74.6% and 41.4%, corresponding Ra values of 81 nm, 45 nm and 37 nm, respectively. Therefore, it can be concluded that the defect density in SLG increases with increasing Ra. In other words, the flattest Cu surface (Ra ≈ 37 nm) has the lowest defect-containing SLG coverage after oxidation testing. This result confirms that surface roughness can degrade the structural stability of SLG during thermal oxidation, but then raises important questions as to why and how this degradation occurs. To reveal the mechanism for the difference in the defect densities with different surface roughness, a DFT calculation was used to analyze the oxidation of SLG on different Cu substrates. Considering that surface roughness consists of surface steps, SLG-coated absolute flat Cu (Figure 5b) and SLG-coated stepped Cu (Figure 5c) were constructed. The DFT calculation showed that the formation energy of a C-O bond on the absolute flat SLG/Cu surface is 1.05 eV. By contrast, the formation energy of a C-O bond on a stepped SLG/Cu surface is 0.19 eV, which is ~82% smaller than that on the flat surface. The reason for the difference in formation energy may be attributed to the increased localized strain in the stepped region [39,58]. These results suggest that the formation of C-O bonds on the stepped SLG/Cu surface is more energetically favorable. In addition, the density of O atoms on the step region was found to be high, which is consistent with experiments showing that the O content appeared to be higher on the rough Cu surface (Figure 4). Therefore, combined with this experiment and the DFT calculation, the lower formation energy of C-O is assumed to be the main factor determining the structural stability of SLG on rough Cu surfaces.

In summary, a 500 °C high-temperature treatment was conducted on single-layer-graphene/copper (SLG/Cu) foil with similar SLG qualities and various copper surface roughness. Raman spectra analysis showed the presence of high densities of sp3 defects in SLG after oxidation treatment. As the Cu surface roughness decreased from 81 nm to 37 nm, the density of the D peak decreased by 40%, and the O content in the SLG/Cu foils decreased from 51.5% to 18.3%, suggesting that the formation of sp3 defects is likely related to Cu surface roughness. Combined with DFT calculations, this study confirmed that the lower formation energy of the C-O bond on rough Cu surfaces (0.19 eV) is one of the influencing factors that may reduce the high-temperature antioxidation property of Gr/Cu. The present results not only enrich our understandings on the degradation mechanism of Gr on Cu, but also suggest a potential strategy to enhance the oxidation resistance of Gr coated metals by reducing the surface roughness of metal substrate.

## Figures and Tables

**Table 1:** The details of the designed samples.

Treatment Conditions	Samples		
Synthesis of SLG on Cu	SLG/Cu		
Re-annealing at 600 °C	A-30 (30 min)	A-60 (60 min)	A-90 (90 min)
500 °C oxidation for 60 s	O-A-30	O-A-60	O-A-90

## Data Availability

The data in this study are available upon request.
